# DNA stability: a central design consideration for DNA data storage systems

**DOI:** 10.1038/s41467-021-21587-5

**Published:** 2021-03-01

**Authors:** Karishma Matange, James M. Tuck, Albert J. Keung

**Affiliations:** 1 Department of Chemical and Biomolecular Engineering, North Carolina State University, Raleigh, NC USA; 2 Department of Electrical and Computer Engineering, North Carolina State University, Raleigh, NC USA

**Keywords:** Synthetic biology, DNA computing and cryptography

## Abstract

Data storage in DNA is a rapidly evolving technology that could be a transformative solution for the rising energy, materials, and space needs of modern information storage. Given that the information medium is DNA itself, its stability under different storage and processing conditions will fundamentally impact and constrain design considerations and data system capabilities. Here we analyze the storage conditions, molecular mechanisms, and stabilization strategies influencing DNA stability and pose specific design configurations and scenarios for future systems that best leverage the considerable advantages of DNA storage.

## Abstract (web summary)

DNA has the potential to store vast amounts of data but it is subject to physical decay. In this Perspective, the authors propose that the stability of DNA should be a key consideration in how it is used for data storage.

## Introduction

“My hunch is—and I’m not alone in this—that the next decade or so will see this used technically. The machine could be much smaller; it could carry a much larger set of data.” In 1964, just 7 years after the discovery of the structure of DNA^1^, Wiener and Neiman discussed the potential density advantage of using nucleic acids as a form of memory storage^2^. Over half a century later, advancements in our understanding of the properties of DNA have confirmed its high theoretical information density of nearly 455 billion GB of data per gram^3^, ~6 orders of magnitude greater than even the most advanced magnetic tape storage systems^4^. DNA also provides a host of other unique potential advantages including highly parallelized computation within the storage system itself^5,6^, low energy requirements^7–9^, rapid high capacity transportation of data^10^, potentially longer lifetimes, and stabilities of decades or centuries compared to conventional media which are replaced every 3–5 years, as well as ease of replication^11^ by molecular biology approaches to ward off degradation. While it has taken more than just the decade Wiener predicted, a large body of knowledge in molecular biology^12,13^ and computer and information systems^14,15^ has been developed in the intervening period that has set the foundation for the recent interest and investment in DNA-based information storage technologies.

The nature of Wiener’s ‘machine’ will remain in constant flux and development. Indeed, multiple types of systems may arise to address different applications, from long-term ‘write-once-read-never’ archival storage to highly dynamic and frequently accessed data storage, potentially with in-storage computational capabilities. It is important to imagine these possible types of DNA information storage systems and the different unit processes that will comprise these systems, and identify how the chemical, physical, and encoding properties of DNA will influence their design. As DNA or an analog will be the substrate of this class of polymeric systems, its stability under different environmental and process conditions will be a central design consideration, informing the nature of both physical unit processes and encoding algorithms.

**Fig. 1:**
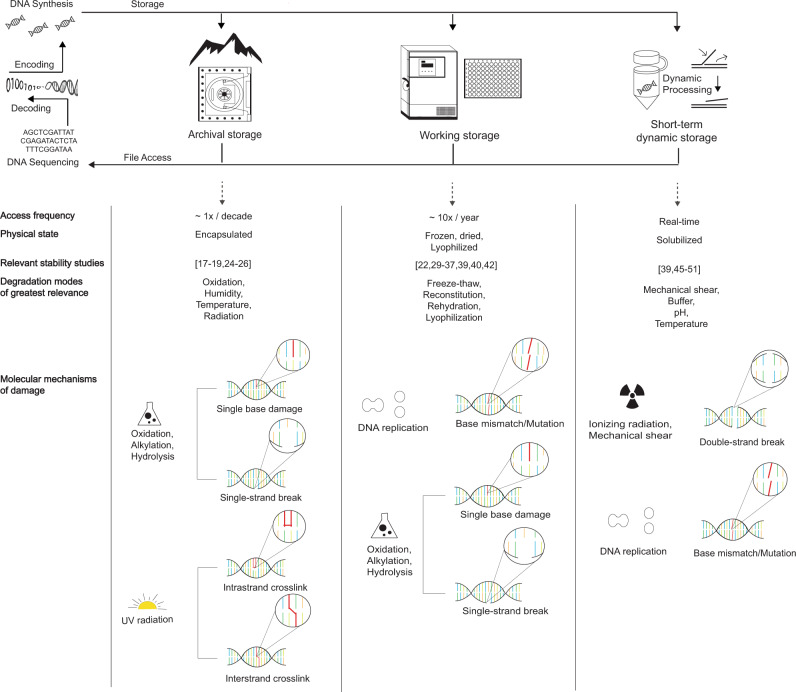
Categories of DNA storage with distinct longevities, functional characteristics, and degradation modes. (Top) A generic DNA-based system showing distinct types of storage modes. (Middle) Functional and physical characteristics of each storage mode. (Bottom) Molecular mechanisms of damage most relevant to each storage mode.

An end-to-end DNA storage system is depicted in Fig. 1 with generic unit processes. As applications range from cold archival storage to frequently accessed or even dynamically manipulated data, the DNA is exposed to more manipulation such as phase changes or physical shearing through liquid handling, and to more distinct types of environmental conditions such as buffers with different salt concentrations and pHs. These present more opportunities for degradation as well as specific degradation mechanisms that may influence encoding strategies and sources for data and decoding errors (Fig. 1). Here we review what is known about the stability of DNA under each of these conditions, organized by their relevance to systems with different operating timescales. We then provide a quantitative analysis of the relative tradeoffs in density, physical redundancy, and encoding strategies that must be sacrificed to achieve increasingly sophisticated system capabilities such as increased access frequency and in-storage computation.

It will be useful to also describe the molecular architectures common to almost all DNA storage systems proposed to date. DNA storage systems are comprised of many files, and each file consists of many distinct DNA strands that typically are ~150–200 nt long as that is the current limit of phosphoramidite synthesis chemistries, but could be longer with advances in technology. All strands comprising one file share a common address sequence located on one or both ends of the strands. These addresses can be read and retrieved using PCR or transcription. Mutations within strands, or loss of strands due to breakage or degradation would lead to potential decoding errors or even complete loss of information. Therefore, error correction codes are usually applied to help compensate for errors and lost strands with the trade-off of decreasing information density.

## Write-once-read-never archival storage (access frequency once every ~10+ years)

One of the most likely first applications for DNA-based information storage will be long-term ‘cold’ storage intended for the preservation of historical or other records across decades or centuries. This is because the current high cost of DNA synthesis and sequencing can be justified when they are infrequent and amortized over many years^16^. What is crucial for these applications is a strong understanding of the long-term stability of DNA.

The successful recovery of DNA from fossils or microbes preserved in permafrost, some millions of years old, is commonly used as motivating evidence for the long-term stability of DNA^17,18^. However, there are several caveats to consider. First, there are substantial concerns that some of these results were misinterpreted due to potential contamination from contemporary bacteria or human DNA^19^. Therefore, the current best estimate for the recovery of DNA from natural samples based upon a rough agreement between theoretical calculations^19^ and physical measurements^20^ is roughly 400,000 years, with the major degradation mechanism in these samples thought to be cross-linking between strands that inhibit PCR-based amplification and detection of the DNA.

Second, estimations of DNA stability from natural samples may be overly optimistic for DNA storage applications. In the context of recovering DNA from fossils, ribosomal and mitochondrial DNA are often used to identify different species of organisms but can be present at hundreds of copies per cell. Yet, these are the sequences used when claiming successful isolation of DNA when in fact genomic DNA sequences are much more difficult to recover than multi-copy mitochondrial DNA. Thus, while some DNA can be recovered from fossilized or permafrost samples, often only the most abundant sequences are detectable due to substantial degradation. This is further reflected by the estimated half-life of fossilized DNA being only ~500 years, corresponding to a per nucleotide fragmentation rate of 5.50E−6 per year assuming an average 242-nt long mitochondrial DNA sequence in the relatively cold temperatures of permafrost (5.5E−6/nt/yr ∗ 242 nt ∗ 500 yrs ~ 50%)^17^. This is substantially lower than the estimated 400,000 years estimated to recover any DNA signal. Thus, in the context of DNA storage applications where a substantial fraction of the data should be recoverable depending on the encoding strategy and amount of physical redundancy, the ‘useful’ stability of storage systems, based upon data from fossilized DNA, suggests stabilities of a few hundred years or less.

While there are likely substantial stability limitations of natural permafrost or fossilized samples, and frozen aqueous samples in general, fortunately DNA storage systems can be engineered with highly controlled materials and environmental conditions that could substantially augment its stability. Several approaches have been tested, most involving dehydrated forms of DNA to reduce hydrolysis of its phosphate backbone. For example, DNA has been adsorbed onto Flinders Technology Associate (FTA) filter cards, stored in biopolymeric storage matrices such as the commercial product DNA Stable, embedded into silk matrices, or simply stored as lyophilized powder^20,21^. In many cases, the adsorption of the DNA onto a matrix stabilized the DNA. For example, over 40 days at 25, 37, and 45 °C, 80% of the DNA embedded in silk was recoverable compared to 20% when unprotected^22^. Silk also offered protection against UV radiation. Salts have also been shown to offer stabilizing effects for dried DNA particularly against high ambient humidity and can maintain high loading of DNA (>20 wt%) while keeping the DNA relatively accessible^23^.

Of the many different methods tested, the current leading approach that consistently exhibits the best stability has been encapsulation of DNA within an inorganic matrix comprised of silica, iron oxide, or a combination of both. Multiple groups have shown that encapsulation can substantially enhance DNA stability^24–27^. For example, Puddu and colleagues directly compared the stability of encapsulated DNA with unprotected DNA at 100 °C for 30 min. Eighty percent of the encapsulated DNA was recovered while only 0.05% of the unprotected dried DNA survived. Grass and colleagues estimated that encapsulation in silica particles could maintain DNA for 20–90 years at room temperature^25^, 2000 years at 9.4 °C^24^, to over 2 million years at −18 °C. These estimates were derived from accelerated aging models applied to data obtained from DNA exposed to elevated temperatures of 60–70 °C. In a few studies, diverse populations of DNA strands encoding actual data were decoded after accelerated aging, providing estimates not only of half-lives, but also evidence that complete files could be retrieved^24,25^. In several cases, data could be retrieved and re-embedded multiple times, although degradation was observed. ‘Break points’ in these systems would depend on the physical redundancy as well as density overhead sacrificed to enable degradation-tolerant encodings.

This work provides substantial evidence for the long-term stability of DNA, especially encapsulated in silica. However, there are several potential limitations to consider. First, the physical processes of encapsulation and retrieval take some time, suggesting this approach is best suited for cold storage applications. Second, the encapsulation of the DNA inherently reduces the information density of the storage system. A layer by layer design with alternating DNA and cationic polyethylenimine with a silica final encapsulation has achieved the best storage density to date in such systems, ~3.4 wt% DNA^25^. This is a sacrifice of 1–2 orders of magnitude in information density; yet, while not as dense as pure DNA, given the 5–6 orders of magnitude advantage of DNA storage over conventional storage media, sacrificing 1–2 orders of magnitude of density would still yield a very space-efficient system.

The size of DNA strands is also an important design consideration. One hope in the DNA storage field is to develop synthesis technologies that are able to create longer DNA strands. The benefit of longer strands would be to reduce the percentage of overhead per strand devoted to file addresses and indices, thereby increasing the information density of the system. However, prior work has already suggested diminishing returns with regards to density with increasing strand length^3^. Further supporting the use of shorter DNA strands, empirical evidence suggests that shorter strands are more resilient to environmental insults. For example, exposure to 830 W/m^2^ of sunlight irradiation led to nearly 2 orders of magnitude greater degradation for a 113 nt compared to a 53 nt DNA strand. While constant exposure to UV is unlikely for a DNA storage system, it may reflect a general principle of length-dependent degradation sensitivities. Indeed, other environmental insults exhibit similar length dependencies, with exposure to 90 °C thermal treatment leading to nearly 3 orders of magnitude greater degradation for longer strands^28^, while shorter strands were more resilient to freeze-thaw cycles.^29^ As these are accelerated degradation studies, it is likely that longer strands will be sufficiently stable for practical use; however, shorter strand lengths would likely provide improved stability profiles.

Finally, it is important to note that while seemingly simple, accurately determining long-term DNA stability is not trivial nor a solved problem. There are two primary challenges. First, the accelerated aging models used in many studies that can lead to stability predictions of hundreds or even millions of years are inherently extrapolations and could therefore deviate from actual stabilities over long periods of time. This is especially true if there are unknown degradation mechanisms that are important over long timescales but that do not exhibit the exponential dependency on temperature specifically as commonly assumed by many models. Given that the accuracy of data retrieval is paramount for cold storage systems, additional studies assessing accelerated degradation due to other parameters other than temperature would provide increased confidence in extrapolated stabilities. For example, a broader variety of storage conditions should be tested to develop a more comprehensive mechanistic model of DNA stability, including models that not only artificially accelerate aging through elevated temperature but through elevated exposure to electromagnetic radiation and/or subatomic particles, high or low pH, hydrolysis and humidity, and mechanical rearrangements (e.g., freeze thaws, liquid handling) at the molecular level. In addition, DNA concentration and encapsulation conditions could have inherent effects on stability as there could be degradation mechanisms arising from chemical interactions between DNA molecules or between DNA and the encapsulating materials.

A second challenge of accelerated aging studies is that methods to measure degradation often are not sensitive enough, so rely on large degradation effects or require amplification steps (e.g., through PCR) that could skew or bias results. These issues motivate new future gold standards to assess long-term stability. New approaches could include a combination of deep next-generation sequencing of short-term samples to detect very rare degradation events. In addition, it may be advisable for the field to collaboratively initiate real time, non-accelerated, long-term studies of DNA stability over the course of a human generation or more, and compare these results to deeply sequenced short-term studies as well as those using different modes of accelerated degradation. These could be performed on actual commercially active storage systems as both a way to monitor stability and to provide insights into long-term stability. This work could be paired with studies of different environmental insults to identify dominant degradation modes and their relative contributions to aging. Overall, this data could be used to update and benchmark the stabilities of any DNA storage systems that were created in the past, and to continually inform and adjust models predicting their stability.

## Working storage (~ accessed multiple times per year)

While there is promise for the ability to store DNA stably over centuries or even millennia, methods capable of this type of stability typically require fully encapsulating and sealing DNA within a matrix, like silica. This is because encapsulation protects the DNA from exposure to humidity, radiation, fluctuations in temperature, and other potential reactants. Furthermore, it may be the most robust and economical storage method, requiring the least amount of specialized storage equipment such as tightly controlled refrigeration and humidity. However, due to substantial DNA loss associated with retrieval from encapsulated media, and the relatively involved encapsulation and retrieval processes, this form of storage is best matched with applications where access is infrequent or never occurs. In a generic DNA storage system, encapsulated DNA could be used to store the ‘master’ or preservation copy of data, while ‘working’ copies are maintained using less stable but more accessible methods.

There are three semi-accessible forms of storage that might be compatible with working storage, all of which are similar to how research labs in the biological sciences currently store DNA samples: refrigerated in aqueous solution, frozen in aqueous solution (typically at −20 or −80 °C), or as a dry solid. While DNA recoveries near 100% have been reported from all of these storage forms after ~2 years^30–33^, accelerated aging studies at elevated temperatures suggest storing DNA in these forms would not be sufficient for multi-decade stability^21,34^ especially when compared to encapsulated storage strategies. For example, at 70 °C, substantial degradation of dried, adsorbed, and aqueous DNA was observed at 15–70% after only 5 days^22,23,35,36^, while encapsulated DNA showed no appreciable degradation after 7 days^22^. This may be surprising to many working in the biological sciences where plasmid DNA preparations are stored for entire scientific careers; however, it is important to consider that many such research samples are usually single plasmid constructs stored at very high copy number (10^10^ copies/μL − 1 μg/μL) so that recovery of the plasmid through bacterial retransformation can occur even with 99% DNA degradation. Regardless, there is strong evidence that storage at 4 °C or below in aqueous or dried form would provide at least a couple of years of stability, appropriate for storing working copies of DNA-based information^31–33^, with lyophilized DNA showing better stability than aqueous DNA solutions^37^. One additional important caveat for DNA solutions appears to be the starting concentration of DNA, with dilute solutions of ~0.02 ng/mL exhibiting substantial degradation within weeks when stored at −20 °C^30^. Inclusion of non-specific carrier RNA or DNA was able to abrogate this degradation.

If stored for only a few years, the key concern for working copies of data then becomes the amount of degradation that occurs each time information is accessed, e.g., freeze-thaw of solubilized DNA or rehydration of dried DNA. Likely due to the ability to directly measure the effect of multiple access events within a reasonable experimental time frame rather than needing to simulate or accelerate degradation over time, many more studies have investigated these mechanical effects on DNA stability as opposed to the basal stability of unperturbed samples.

During the freezing and thawing process, ice crystals are formed that generate forces that could lead to breakage of DNA polymers. There is also evidence that freezing makes DNA more susceptible to breakage than non-frozen DNA at similar tensional forces^38^. Several studies have repeatedly frozen and thawed DNA samples, generally observing exponential degradation. An approximately 10% degradation of lambda DNA in Tris-EDTA buffer was observed after 1 freeze-thaw, and 75% degradation was observed after 20 freeze thaws^39^. An exponential fit modeled this process relatively closely (% intact DNA = 0.9484*e^−0.068*freeze-thaws^). A study of DNA stored in water or 50% glycerol found similar degradation rates with more than 75% degradation after 16 freeze thaws, and samples in 50% glycerol performing worse than in water^40^.

As with archival storage, one important consideration in assessing degradation from freeze thaws is the size of the DNA strands. Empirical evidence suggests smaller DNA strands would be preferable for improved stability against freeze thaws. This effect was observed comparing genomic DNA above and under 100 kb, but also at smaller length scales of 5 kb^29^. In addition, increased DNA concentrations exhibited a self-protective effect. As freeze thaws cause mechanical stresses on DNA, there is some intuition for why longer DNA strands would be more susceptible to breakage. The effect of concentration of DNA stability may be due to less intuitive mechanisms that should be investigated in more detail, such as altering the phase transition boundaries of the aqueous solution^41^.

Dehydration is another means of storing DNA at intermediate (~2–10 years) timescales. Vacuum dried DNA, for example, was shown to be stable at 5 °C for at least 22 months^36^. While detailed studies of repeated rehydrations are lacking and need to be performed, a few studies have assessed the recovery efficiency from dried DNA and the impact of prior exposure to elevated temperatures on this recovery. For example, a library of 2042 unique sequences comprising ~20 kb of data was dehydrated on a microfluidic “PurpleDrop” device^42^. Rehydration with water was performed at various dwell times ranging from 1 to 120 s. While increasing the dwell time generally increased the amount of DNA recovered, just 1 s was able to recover ~77% of the DNA by mass. The degradation rate was not directly reported but with sufficient physical redundancy (copy number) and sequencing depth, the entire file was recovered and successfully decoded. Of note, the consistency of the retrieval process exhibited substantial variability and suggests further work investigating mechanisms of the dehydration process, potential irreversible denaturation of the DNA into different helical forms (i.e., forms A or B), and molecular degradation chemistries will be important. For example, exposure of dried DNA to elevated temperatures could irreversibly denature the secondary helical structure of DNA^35^, resulting in samples that are difficult to process or sequence.

Other forms of DNA instability should also be considered for both solution and dried sample storage methods. These include depurination (removal of adenine or guanine bases from the DNA backbone) and oxidation (8-oxo-dG). Both in solution and in dried form, the rate of depurination and oxidation are both higher by 1–2 orders of magnitude compared to strand breakage. Roughly 6% of strands that are 200 nt would develop a depurinated or 8-oxo-dG nucleotide per year at room temperature^35^, with rapid increases in these rates with temperature, following an Arrehenius dependence. These degradation mechanisms have functional impacts. Depurination would result in missing or incorrect nucleotides during sequencing, while 8-oxo-dG can lead to cross-linking between DNA strands which inhibits both rehydration of dried DNA as well as amplification and sequencing of the DNA. Depending on the encoding method and physical redundancy of the storage system, these could further reduce the estimated longevity of solution, frozen, and dried storage methods.

## Short-term storage for dynamic handling of data

The active development of DNA-based computation preceded the development of DNA storage systems by over a decade^5,6,43,44^. The idea of now merging these two fields to perform in-storage computation has intriguing implications including potentially providing the ability to directly search^45^ and edit^46^ DNA databases. In addition to computation, there is also the hope that DNA storage systems may be capable of dramatically lower latencies of operation than the current multi-hour to multi-day process of reading and writing information. For both of these applications, physical manipulation of DNA will be necessary. An important factor to consider for dynamically accessible storage is the complexity of storage methods. Most likely, DNA for these applications will need to be stored in a soluble aqueous form with buffers compatible with molecular processes including dynamic DNA–DNA hybridizations, transcription or polymerization, and other enzyme-driven reactions. Alternatives may include the use of magnetic particles as long as the adsorption/desorption of DNA is relatively rapid. Understanding and potentially improving the stability of DNA in these contexts will inform the operating lifetime of systems as well as dictate the types of error-tolerant encodings that will be necessary.

Almost all manipulations performed on DNA will involve liquid handling, often through small apertures such as pipette tips, microfluidic channels, or tubing. These manipulations impart shear forces on DNA that can lead to strand breakage. Several studies have investigated the effect of vortexing or the use of other shearing devices on DNA stability and showed substantial degradation, for example nearly 70% fragmentation after 3 min^39,47–49^ on a standard table-top vortexer at maximum speed. While these types of shearing devices are unlikely to be used in DNA storage systems, they may be useful in providing relationships between degradation rates and shear forces or energy inputs if used in controlled settings, like rheometers. More directly relevant to storage systems is the measurement of DNA fragmentation due to actual pipetting. With a relatively rapid single pipetting action through a 1 mL tip, 70% of long lambda phage DNA was fragmented. Slower and gentler pipetting  still led to more than 50% of lambda DNA being fragmented^39^. This is somewhat concerning given liquid handling often uses even smaller 200, 20, and 2 μL tips that would result in higher shear forces. With automation, the pipetting actions could be slowed significantly to be less turbulent; however, this might present a significant trade-off in increasing the time required to perform each physical operation or manipulation. What is clear is whatever form of liquid handling is used, careful assessment of DNA stability will be important and processing parameters tuned to ensure DNA stability. New forms of liquid handling should also be explored but also quantitatively assessed for their impacts on DNA stability. For example, acoustic liquid handling of nanoliter to microliter droplets, may exhibit distinct impacts on DNA stability through additional types of forces such as exposure to surface tensions through high surface area to volume ratios. In addition, as in the case of freeze-thawing where smaller DNA strands were qualitatively shown to be more resistant to fragmentation, comprehensive determinations of thresholds and quantitative relationships between DNA length and resiliency to shear forces are currently lacking but would be useful for designing storage systems with low latency or in-storage computation.

While physical manipulations will likely impart some level of unavoidable damage to DNA, the biochemical environment of the DNA and the properties of the DNA molecules themselves could improve stability and resilience to these processes as well as enhance stability in aqueous and unfrozen states. For example, as was already discussed, DNA stored at 4 °C can remain relatively stable over a few years, and maintaining a high concentration of DNA or including carrier DNA or RNA helps slow down degradation rates. DNA degradation can occur through several mechanisms including oxidation and hydrolysis of the phosphate backbone or the base from the sugar (depurination). In addition to temperature which generally accelerates rates of most degradation reactions, controlling pH is perhaps the next most obvious candidate for improving DNA stability against these mechanisms. Both acidic and basic conditions can enhance the hydrolysis rate of DNA by either increasing the electrophilicity of the DNA or the nucleophilicity of water. For example, it was estimated that a change from pH 6 to 5 could increase the degradation rate of DNA by an order of magnitude^50^, and even just a 90-min exposure to pH 4.0 at 65 °C led to almost complete degradation of DNA samples^51^. While these experiments were performed at elevated temperatures that might be argued could be avoided, there are many molecular biology manipulations that require transient exposures to elevated temperatures above at least 37 °C and often above 70 °C where many polymerases function optimally. These include DNA in-storage computation, in-storage editing, or even simply copying information by PCR. Furthermore, for search-type functions that rely on DNA–DNA hybridizations, stable hybridizations for ~20 nt sequences occur at between ~50 and 60 °C. Room temperature operation would not provide adequate energy to first melt any secondary structures that might have formed that would block hybridization, and would also result in non-specific sequences hybridizing with each other. Shorter DNA sequence lengths that hybridize near room temperature would be too short to confer adequate sequence specificity amongst large, highly diverse libraries of DNA strands that will comprise most DNA storage systems. Therefore, tightly controlling pH as well as the time exposure to elevated temperatures will be important design considerations for storage systems. The use of many standard buffers such as PBS and TE could help maintain pH levels at appropriate targets. Other additives may also have protective effects including DNAstable^TM^ ^52^, salts^23^, and trehalose^21,30^.

## Tradeoffs between DNA stability and information density

**Fig. 2:**
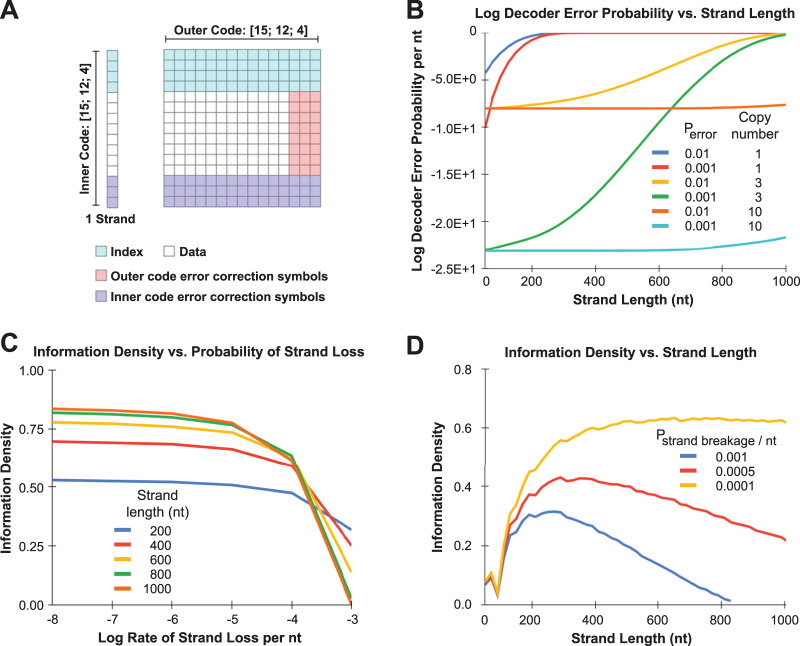
Analysis of tradeoffs between system reliability, information density, strand length, and error rates. **A** Reed-Solomon inner-outer encoding scheme. **B** Relationship between log decoder error probability during RS decoding and DNA strand length, including the effects of symbol error rate (mutations, insertions, and deletion, *P*_error_) and copy number. **C** Relationship between information density of a DNA storage system and the probability of symbol erasure (strand loss due to breakage) as a function of strand length. **D** Relationship between information density and strand length as a function of the probability of strand breakage. **C** and **D** assume a copy number of 1.

This review has focused on empirical measurements of DNA stability under a range of different conditions. Together with theoretical analyses^4,53,54^, there is strong evidence for the utility of DNA as a storage medium. Nevertheless, DNA has finite stabilities, and is especially labile in conditions relevant for frequent access and dynamic processing. This does not preclude the use of DNA for storage applications but does affect the information density of systems by requiring higher redundancy in the number of copies of each distinct strand as well as in the encoding methods used to achieve certain reliability. Understanding the effect of DNA stability on these tradeoffs will be important in designing unit processes and systems for specific types of storage applications. Here we discuss these tradeoffs through a series of analyses shown in Fig. 2, and in particular demonstrate how relationships between copy number, strand loss, strand length, information density can be modeled and used to inform system design. The goal here is to show how models can reveal both intuitive and unintuitive relationships between parameters; it is important to note that ranges of parameter values and observed trends shown here may change depending on the specific details of each system.

Most DNA data storage systems use short sequences or ‘addresses’ written into strands of each file for retrieval. These addresses typically are complementary to short DNA oligomers used to amplify the files through PCR^3^ or to extract them through affinity-based methods^55^. To function at reasonable temperature ranges (<100 °C) the addresses are generally limited to <25 nt as longer sequences would require higher temperatures to ‘melt’ during PCR cycling. However, addresses cannot be much shorter without sacrificing diversity in addresses^46^. The addresses take up space on each strand and do not encode data (e.g., overhead). Index sequences are also overhead and are necessary to include to know how the different strands comprising a file should be ordered.

In addition to the address and index, error correction codes may use additional overhead. Electronic storage systems adopt and employ many error correction mechanisms to ensure the reliability of stored data. Similarly, to cope with frequent errors, DNA storage systems can leverage error correction codes that are capable of detecting and correcting errors that occur as a result of strand breakage or loss and due to substitutions, insertions, and deletions within strands. Error correction codes work by adding enough redundancy to recompute the original data even in the presence of errors or missing strands. Hence, to maintain the same reliability of information transfer or recovery, error correction forces a trade-off between density of information and tolerance to errors. The higher the likelihood of strand error or loss due to reduced DNA stability, the more overhead must be spent on error correction.

While a variety of codes have been proposed for DNA storage, Reed-Solomon (RS) codes are particularly popular given their configurability and tolerance to errors^24,56–58^. The error correction properties of RS codes are well known, and we use them here to explore the combined effects of strand errors, breakage, and length on the information density and reliability of DNA storage systems. We will not focus on the details of different codes but rather use RS codes to illustrate some general trends to consider when designing DNA storage systems. Additional details of popular error correction approaches can be found in a few recent references^14,15^, while details about our implementation of RS codes and parameters used (shown in brackets) can be found in the Supplemental Information. In brief, the key features of the implementation used here are (1) that the RS code is capable of detecting and differentiating two kinds of errors, symbol errors such as insertions, deletions, and mutations, collectively having a probability of *p*_error_, and symbol erasures such as the breakage or loss of a DNA strand with probability *p*_strand erasure_; (2) inner and outer RS codes are interleaved where the inner code protects against errors within a strand and the outer code corrects for missing or erroneous strands (Fig. 2A); (3) an error probability can be calculated to estimate the probability the code will not be able to account for and correct errors given certain error and strand loss rates; (4) the amount of redundancy in the code is tunable in order to achieve a certain error probability or system reliability; (5) the code accounts for multiple copies of each distinct DNA strand, with strand loss or ‘erasure’ only occurring when all copies of a distinct DNA strand are lost; and (6) the length of the DNA strands can be tuned with effects on density due to the ‘overhead’ of addresses and indices.

### Decoding error analysis

To provide a non-exhaustive example of the potential importance of trade-off analyses, here we focus on a major mode of DNA degradation, strand loss through hydrolysis or mechanical breakage. First, to better understand the impact of strand loss on the probability of a decoding error, we analytically model its effect on the decoding error probability of an outer RS[255,223,33] code. Based upon experimental observations, we conservatively model the probability of strand breakage as linearly dependent on strand length, and we assume that strand loss due to breakage is equivalent to an erasure in the outer code. We further assume that multiple copies of a strand exponentially reduce the likelihood of loss because all copies of the strand must be lost to cause an erasure. An exact analytical formula for this probability is unknown so we estimate the relationship as *p*_strand erasure_(length *L*, copies *c*) = (*L* ∗ 5*E*−3)^*c*^. This equation is chosen so that strands of length 1000 nt have a probability of 50% or less of erasure, a value that can be tuned depending on the stability measured for any particular DNA storage system. We fix the *p*_error_ to 1e−2 and 1e−3 per nt to cover the range of typical synthesis and sequencing errors^59,60^.

Figure 2B shows the impact of longer strands on the decoder error probability for several system configurations with different copy numbers of each strand and different error rates. The *y*-axis shows the log decoding error probability and the *x*-axis is the strand length. We have chosen for comparison a system with similar read error rates to hard drives (on the order of 10^−14^ per bit, log error probability of −1.5E + 01)^61^. For this system that has a linearly dependent probability of strand breakage on strand length, the trend is that longer strands increase the likelihood of strand loss, and substantially increase the decoder’s probability of error. Other parameters matter, too. Lower *p*_error_ significantly reduces the residual likelihood of error. Also, higher numbers of copies per strand also have a large effect since it becomes exponentially less likely that all copies of a strand are lost. Current experimental systems tend to operate in a regime with large numbers of copies; however, even if future systems may wish to be more lean, this analysis suggests copy number requirements may not need to be as high as intuition may suggest particularly if strand length designs remain in the 200–500 nt range.

### Information density

To demonstrate how the relationship between strand loss and information density can be studied, we vary the probability of strand breakage per nt from 10^−3^ to 10^−8^ and analyze the information density for many different strand lengths. For a given strand length, there are many different possible designs for a RS inner-outer code. We use an algorithm to find a design with a residual error probability <10^−14^ and maximum information density while keeping the outer block size at 255, and the index to 4. In Fig. 2C, we report the density on the *y*-axis vs. the probability of strand loss on the *x*-axis for each strand length (*L*) considered. We assume a single copy of each strand to maximize information density and fully exploit the error-correcting capability of RS codes.

In this system, shorter strands achieve superior density at high strand breakage rates. This is a result of shorter strands being overall less likely to break, and therefore the outer code needs fewer error correction symbols. Longer strands have a higher overall likelihood to break and need more error correction in the outer code to compensate. However, as the overall probability of breakage per nt decreases (to the left), all strands are less likely to break, and this gives longer strands an advantage since they can hold a larger fraction of information per strand and the outer code can work effectively even with a relatively small number of error correction symbols. A deeper analysis further shows that the magnitude of the strand breakage rate can fundamentally alter the relationship between information density and optimal strand length (Fig. 2D). In fact, it is not simply that shorter strands are better for high strand loss rates but that there can be optimal lengths that balance the overhead needed for file addresses and indices with the encoding overhead required to account for strand loss.

This analysis is just one example of many that can be performed interrogating the effects of diverse parameters of DNA storage systems. It underscores the need to tailor error correction and system parameters like strand length and copy number to different settings including error rates that vary according to environmental conditions and data storage applications. For example, long-term archival storage will likely encapsulate the data in silica helping keep the probability of strand loss or breakage low, allowing longer DNA strands to be used and achieving higher information densities. In contrast, working storage or short-term dynamic storage would benefit from shorter strand lengths to compensate for higher strand loss rates.

## Future prospects

The robustness and failure rates of information storage systems are of utmost importance as the reliability of data retrieval must be concretely reported, verifiable, and trustworthy^62–65^. While we have some rough estimates and measurements of DNA stability in a variety of conditions, often measurements exhibit considerable noise and variability between experimentalists and research groups in addition to substantial noise between samples in an individual experiment. There are likely experimental details affecting the accurate interpretation of measurements including the manipulation of DNA itself in setting up experiments, or confounding parameters like DNA solubility. Experiments exploring a more comprehensive set of parameters and that assess sources of variability in results could provide more confidence in the design and utility of DNA storage technologies. Fine-tuning exact buffer conditions, assessing changes in its composition, and maintaining strict control and provenance records over the environmental exposures of the DNA throughout its complete lifetime starting with DNA synthesis will be important for commercial DNA storage products.

In addition, while many studies have quantified DNA degradation through some form of quantitative PCR, mass measurements, or even next-generation sequencing, the definition of DNA degradation remains unprecise and too limited despite its considerable impact on the physical design and encoding of reliable storage systems. For example, there are many ways a DNA storage system can be degraded. (1) The loss of DNA strands could be biased toward strands with certain properties such as length, base content, or presence of specific sequences. (2) DNA strands may be fragmented in different patterns and frequencies depending on base content, length, and physical processing conditions. (3) Depurination or chemical alterations of bases may occur and not be directly assessed by sequencing or QPCR based approaches. How each of these types of degradation mechanisms are affected by environmental conditions is important for system design and should be carefully assessed.

There is also the intriguing possibility that the physical stability of storage systems could be enhanced by the use of another polymer that is chemically more stable than DNA, although substantial work would likely be needed to replicate the synthesis, processing, and sequencing technologies and infrastructure available for DNA^66^. For example, ‘locked’ nucleic acid monomers possess a methylene bridge between a 2′ oxygen and the 4′ carbon of the pentose ring and offer substantial resistance to nuclease enzymes present abundantly in the ambient environment. There are many other potential chemistries of nucleic acid polymer backbones that may offer differing stabilities tuned for specific environmental conditions or applications, including bicyclo-DNA or glycerol-DNA that have altered sugar backbone chemistries^67^ or nuclease resistant nucleic acids^66^. In addition to altering the biopolymer substrate itself, protective additives or even active repair systems similar to those in natural biological systems may improve storage system reliability.

There are clearly many opportunities and an important need to better understand, characterize, and improve DNA stability. While seemingly a straightforward concept, assessing the stability of DNA and making appropriate choices of storage methods is not trivial. DNA stability has been experimentally measured and reported in many diverse ways, including mutational rate, breakage rate per base, and loss of intact DNA strands. Degradation rates have also been reported in a mix of many different environmental, temperature, buffer, and temporal conditions. Furthermore, the functional impact of different types of degradation will depend on the nature of the storage system. For example, mechanical degradation may affect systems that use longer DNA strands compared to shorter strands, degradation rate may be complicated to predict depending on the density of storage systems due to its potential nonlinear dependency on DNA concentration, and some encoding algorithms may sacrifice information density but be more resistant to the loss of strands. However, it is clear that even with our current nascent knowledge of DNA stability, reliable DNA storage systems can already be created with existing technologies. In addition to developing a better understanding of DNA stability, what will be important is the recognition that the appropriate tradeoffs and limitations in system properties and capabilities should be made and can be supported through models. With improving measurements, a better understanding of degradation mechanisms, new technologies, models, and enhanced encoding algorithms, the efficiency of these systems will continue to improve the commercial viability of DNA-based information storage.

### Reporting summary

Further information on research design is available in the Nature Research Reporting Summary linked to this article.

## Supplementary information

Reporting Summary

Supplementary Information

## Data Availability

Plots in Fig. 2 were developed using Excel and custom code is available at https://github.com/jamesmtuck/DNA_stability under a permissive open source license with instructions for installation.
