# Supplementary material for: DNA stability: a central design consideration for DNA data storage systems
Source: Nat Commun. 2021 Mar 1;12:1358. doi: 10.1038/s41467-021-21587-5 (PMC7921107; doi:10.1038/s41467-021-21587-5)
Supplement: Supplementary file 2 — Supplementary Information [file 41467_2021_21587_MOESM2_ESM.pdf]

Supplemental Information

Additional details of the encoding method and analysis. RS codes are linear codes and specified in terms of three parameters, RS[n, k, d]. n is the length of the coded message, k is the original message length, and d is the minimum distance of any two blocks and equals n-k+1. The distance, d, indicates the error correcting capability of the code. It is worth noting that n is restricted to be of the form q^m-1, where q is a prime number and m is an integer greater than or equal to 1, but k or d can be chosen arbitrarily. Genomic DNA is fundamentally a base-4 code, and hence n may be chosen to be one less than an even power of 2 in DNA storage systems.

Error correction techniques usually differentiate two kinds of errors, symbol errors and symbol erasures. An erasure occurs when the decoder has extra knowledge that an input symbol is missing or erroneous. For example, an erasure occurs if a strand is never sequenced and therefore is never provided to the decoder. The decoder algorithm knows the strand is absent and can leverage that to increase the likelihood of correct decoding. Erasure-only decoding is common in electronic file systems where hard disks are often modeled to either succeed or fail as an entire unit.

A symbol error is what occurs in any other case when no such erasure knowledge is present. The input symbol to the decoder differs from what was originally encoded, and the decoder is unaware of the error and must identify that the symbol is incorrect and fix it through the decoding process.

The RS decoder is less capable of correcting symbol errors than erasures. A maximum of d erasures can be corrected, whereas, a maximum of only (d-1)/2 symbol errors can be corrected. Any combination of erasures and errors can be corrected such that $d > 2*e_{sym} + e_{erasure}$, were $e_{erasure}$ is the number of erasures and e_{sym} are all other errors.

An RS code can be designed to tolerate some number of errors by setting the distance parameter, d, large enough. The probability that it fails to correct can be estimated using the decoder error probability of an RS code[56,57,61], given by $P_E([n,k,d], p_{error}, p_{erasure})$, which calculates the probability that the number of errors and erasures exceeds the bound required for correct decoding, namely $d > 2*e_{sym} + e_{erase}$ would no longer hold given the block length, number of error correction symbols, and the probability of errors, p_{error}, and erasures, $p_{erasure}$.

P_E can be estimated by treating the number of errors and erasures as a random variable following a binomial distribution[56,57]. As a reminder, the binomial probability mass function for a random variable X is given by:

$$f(t; n; p) = \Pr(X = t) = \binom{n}{t} p^t (1-p)^{n-t}$$

The cumulative binomial distribution, denoted $F(t; n; p) = \Pr(X \leq t)$, is the summation of $f(t; n; p)$ over the range where $X \leq t$. Then, for an RS[n, k, d] code, and using the substitution $t = \frac{d-1}{2}$:

$$P_E([n, d, k], p_{error}, p_{erasure}) = (1 - F(t, n, p_{error})) + \sum_{i=0}^{t} f(i; t; p_{error}) \times (1 - F(2(t-i); n; p_{erasure}))$$

Simply stated, P_E is sum of the probabilities of either more than t errors or a combination of errors and erasures such that the number of erasures plus twice the errors is greater or equal to the distance, d, of the code.

The decoder error probability is dependent on the likelihood of symbol errors and erasures in the message sent to a decoder. We can estimate p_{error} and $p_{erasure}$ based on previous studies[55,58,62,63]. The probability

of a single base error in a DNA strand is a function of the combined effects of synthesis and sequencing errors and has been measured empirically to be in the range of 10^{-3} to 10^{-2}. For the case of probability of strand loss or breakage, we sweep the likely error ranges predicted from empirical measurements of DNA stability to estimate $p_{erasure}$.

DNA storage systems often use a combination of both inner and outer RS codes. The inner RS code protects against errors within a strand and the outer code corrects for missing or erroneous strands. The block length of an inner code, n_{inner}, and its error correcting ability, d_{inner}, would be limited to the number of symbols that fit in a strand of a given length and the requirement that an index be present within each strand[58,64,68]. The index must be large enough to uniquely identify each strand of a file, and this is on the order of O(log M), where M is the desired number of strands per file.

The outer RS code is formed as a set of n_{outer} strands, wherein k_{outer} strands hold the information and n_{outer}-k_{outer} strands hold the additional error correction symbols required for the RS code. If a strand is never sequenced or discarded due to too many errors by the RS inner decoder, such a missing strand is treated as an erasure by the outer code. If fewer than d_{outer} strands are lost in a block, the RS outer decoder may recover the block provided that no strands contain erroneous data. If some strands are missing and some strands are erroneous, the previously stated relationship, $d_{outer} > 2*e_{sym} + e_{erase}$, also applies to the outer code as well.

Also, DNA storage systems incorporate in each strand an index that indicates which part of a file a strand corresponds to[58,64,68]. The index must be large enough to uniquely partition all of the data for a large file across strands. The index may be considered part of the data with respect to error correction and protected using the inner RS code. However, with respect to information density, it is usually considered overhead. Hence, the information density, or Rate, of the code is estimated as: $(k_{inner} - i)*k_{outer} / (n_{inner} * n_{outer})$, where i is the number of symbols devoted to the index. Figure 3a illustrates this ratio as white data units divided by all of the other symbols in the code. Figure 3c and 3d use this formula to calculate information density assuming a copy number per strand of 1.
